# Supplementary material for: Lack of knowledge of stakeholders in the pork value chain: Considerations for transmission and control of Taenia solium and Toxoplasma gondii in Burundi
Source: PLoS One. 2025 Jul 2;20(7):e0326238. doi: 10.1371/journal.pone.0326238 (PMC12221015; doi:10.1371/journal.pone.0326238)
Supplement: S1 Appendix — (DOCX) [file pone.0326238.s002.docx]

1. **Questionnaires for interview (quantitative study)**

Date of data entry: ………………….

HH Number: ……………………..

Hill/Quarter..........................................

Sub-hill............................................

Commune......................................

GPS location:……………………………….

1. How old are you? .........years
2. What is your gender? A. Male B. Female
3. What is your education level? A. None B. Primary school C. Secondary school D. University
4. What is your occupation? A. Farmer B. Employed C. Unemployed D. Other (specify).........
5. How many people do you have in your household?
6. Have you heard of porcine cysticercosis? A. Yes B. No
7. If yes, where have you heard from it? A. In the Community B. at work C. at School D. Friends E. Other
8. How does the cyst look like? A. Rice grain in the meat B. Rice grain in the tongue C. I don’t know
9. How do pigs get infected with cysts? (multiple options) A. Eating tapeworm eggs containing human faeces B. Mating with infected pigs C. I do not know D. Other (specify)…
10. Have you heard of a tapeworm infection in humans? A. Yes B. No
11. If yes, where have you heard from it? (multiple options)? A. In the Community B. at work C. at School D. Friends E. at hospital F. Other (specify)…
12. How does a person get infected? A. Eating raw or undercooked meat B. Drinking dirty water C. Eating unwashed vegetables and fruit D. I don’t know E. Other (specify)….
13. What are the symptoms of taeniosis? (multiple options)? A. Presence of proglottids/white segments in stools B. Chronic abdominal pain C. I don’t know D. Other (Specify)……
14. Have you heard of cysticercosis infection in humans? A. Yes B. No
15. If yes, where have you heard from it? (multiple options)? A. In the Community B. at work C. at School D. Friends E. at hospital F. Other (specify)……
16. What are the symptoms of cysticercosis infection in humans? (multiple options) A. Epilepsy/seizure B. Chronic headaches C. I don't know D. Other (specify)….
17. How does a human get infected with cysticercosis? A. Eating fruit and vegetables with tapeworm eggs B. Drinking water containing tapeworm eggs C. I do not know D. Other (specify)….
18. Have you seen someone with epilepsy in your quarter or commune? A. Yes B. No
19. If yes, what do you think about the cause of epilepsy? A. Evil spirits (witchery) B. Genetic diseases C. Trauma during childbirth D. Parasitic diseases (cysticercosis) E. Abdominal gas from epileptic people F. I don’t know G. Other (specify)…….
20. Have you heard of toxoplasmosis infection in humans? A. Yes B. No
21. If yes, where have you heard from it? (multiple options)? A. In the Community B. at work C. at School D. Friends E. at hospital F. Other (specify)……
22. What are the symptoms of toxoplasmosis infection in humans? (multiple options) A. Abortion B. Baby with abnormalities C. Stillbirth D. I don’t know E. Other (specify)…
23. How does a human get infected with toxoplasmosis? (Multiple options) A. Eating food infected with cat faeces B. Eating raw or undercooked meat from infected animals C. I don’t know D. Other (specify)….
24. Do you eat pork at the bar/family/during festivities? A. Yes B. No
25. How often do you eat pork at the bar/family/during festivities? A. Daily B. Twice a week C. Once a week D. Once a month E. Occasionally a year
26. How do you prefer your pork to be prepared? (multiple options) A. Boiled B. Roasted C. Fried D. Other
27. How many grams of pork do you eat per meal?
28. Do you think eating infected pigs is a problem? A. Yes B. No
29. If yes, why is it a problem? A. It leads to taeniosis B. It leads to illnesses C. It leads to epilepsy D. I don’t know E. Other (specify)…
30. Do people infected with tapeworm consult medical services? A. Yes B. No C. I don’t know
31. If no, where do they get consultation? A. Stay at home or buy medicine at pharmacy B. Traditional medicine C. Other (specify)….
32. Do people infected with toxoplasmosis consult medical services? A. Yes B. No C. I don’t know
33. If no, where do they get consultation?
34. Do people infected with epilepsy consult medical services? A. Yes B. No C. I don’t know
35. Do people with epilepsy get assistance from traditional healers? A. Yes B. No C. I don’t know
36. If yes, how much do they pay for traditional treatment?

**II. Topic guide for focus group discussions (qualitative study)**

1. **Perceptions of pork tapeworm infections and toxoplasmosis in pigs and humans**

- What do you think about porcine cysticercosis, pork tapeworm infection, human cysticercosis and its association with epilepsy, and human toxoplasmosis?
- What do you think about how pigs get infected?
- What do you think about how humans get infected with pork tapeworm, human cysticercosis, epilepsy, and toxoplasmosis?
- What are the signs of pork tapeworm, human cysticercosis, epilepsy, and toxoplasmosis?
- How do you perceive these infections in humans ( economic costs and health impacts)?

1. **Perceptions of risk behaviour in culinary practices**

- How is pork prepared? (probes….)
- Are there people in the community who eat raw pork?
- How much time do you spend cooking pork?
- Are there people in the community who eat infected pork with cysts?
- What are the consequences (diseases) of eating pork with cysts from pork tapeworm and bradyzoites (cysts) due to toxoplasmosis?

1. **Perceptions of control methods for pork tapeworm infections and toxoplasmosis**

- What do you think of effective control methods to prevent infections in pigs and humans?
- Are patients diagnosed and treated in medical services, traditional healers, and self-medication?
- What are the factors influencing health-seeking routes for treatment?
