# Supplementary material for: Lack of knowledge of stakeholders in the pork value chain: Considerations for transmission and control of Taenia solium and Toxoplasma gondii in Burundi
Source: PLoS One. 2025 Jul 2;20(7):e0326238. doi: 10.1371/journal.pone.0326238 (PMC12221015; doi:10.1371/journal.pone.0326238)
Supplement: S1 Table — (DOCX) [file pone.0326238.s004.docx]

**S1 Table. Participants’ knowledge of *T. solium* and *T. gondii* infections.**

| **Diseases** | **Questions** | **Bujumbura** | **Kayanza** | **Ngozi** | **Total** | **%** | **Chi-square** | **P-value** |
| --- | --- | --- | --- | --- | --- | --- | --- | --- |
| 1. Porcine cysticercosis (PCC) ‘‘Ubupera’’ | **Has heard about PCC** | | | | | | | |
|  | Yes | 176 | 93 | 97 | 366 | 94.8 | 13.3 | 0.001* |
|  | No | 18 | 1 | 1 | 20 | 5.2 |  |  |
|  | **Knew the appearance of cysts** | | | | | | | |
|  | Rice grain in the tongue, meat, and eye | 163 | 89 | 89 | 341 | 93.2 | 1.3 | 0.52 |
|  | IDK | 13 | 4 | 8 | 25 | 6.8 |  |  |
|  | **Knew the transmission of PCC** | | | | | | | |
|  | Yes | 45 | 22 | 49 | 116 | 31.7 | 21.7 | <0.001* |
|  | IDK/wrong answers | 131 | 71 | 48 | 250 | 68.3 |  |  |
| 1. Pork tapeworm ‘‘Igifwana’’ | **Has heard about pork tapeworm** | | | | | | | |
|  | Yes | 169 | 92 | 90 | 351 | 90.9 | 9.0 | 0.011* |
|  | No | 25 | 2 | 8 | 35 | 9.1 |  |  |
|  | **Knew the transmission of pork tapeworm** | | | | | | | |
|  | Eating raw infected pork | 47 | 44 | 42 | 133 | 37.9 | 14.1 | 0.0009* |
|  | IDK/Wrong answers | 122 | 48 | 48 | 218 | 62.1 |  |  |
|  | **Knew the symptoms of pork tapeworm** | | | | | | | |
|  | Proglottids in stools/chronic abdominal pain/constipation/diarrhoea | 28 | 44 | 27 | 99 | 28.2 | 28.9 | <0.001* |
|  | IDK | 141 | 48 | 63 | 252 | 71.8 |  |  |
| 1. Human cysticercosis (HCC) | **Has heard about HCC** | | | | | | | |
|  | Yes | 5 | 7 | 2 | 14 | 3.6 | 5.2 | 0.073 |
|  | No | 189 | 87 | 96 | 372 | 96.4 |  |  |
|  | **Knew the symptoms of HCC** | | | | | | | |
|  | Epilepsy/Seizure/headache | 4 | 7 | 2 | 13 | 92.9 | 1.9 | 0.380 |
|  | IDK | 1 | 0 | 0 | 1 | 7.1 |  |  |
|  | **Knew the transmission of HCC** | | | | | | | |
|  | Water/fruit/vegetables contaminated with pork tapeworm eggs | 2 | 7 | 2 | 11 | 78.6 | 6.9 | 0.032* |
|  | IDK | 3 | 0 | 0 | 3 | 21.4 |  |  |
| 1. Epilepsy ‘‘Intandara= Igikange’’ | **Has seen someone with epilepsy** | | | | | | | |
|  | Yes | 178 | 68 | 49 | 295 | 76.4 | 64.2 | <0.001* |
|  | No | 16 | 26 | 49 | 91 | 23.6 |  |  |
|  | **Beliefs about the transmission of epilepsy** | | | | | | | |
|  | Parasitic diseases/eating pork infected with cysts | 43 | 24 | 19 | 86 | 22.3 | 1.1 | 0.592 |
|  | IDK/wrong beliefs (evil spirits, flatulence gas) | 151 | 70 | 79 | 300 | 77.7 |  |  |
| 1. Human toxoplasmosis | **Has heard of human toxoplasmosis** | | | | | | | |
|  | Yes | 11 | 8 | 15 | 34 | 8.8 | 7.5 | 0.023* |
|  | No | 183 | 86 | 83 | 352 | 91.2 |  |  |
|  | **Knew the symptoms of toxoplasmosis** | | | | | | | |
|  | Abortion/stillbirth/baby with abnormalities | 4 | 8 | 12 | 24 | 70.6 | 10.2 | 0.006* |
|  | IDK | 7 | 0 | 3 | 10 | 29.4 |  |  |
|  | **Knew the transmission of toxoplasmosis** | | | | | | | |
|  | Eating food infected with a cat (water, fruit, vegetables)/raw meat/transplacental transmission | 1 | 6 | 6 | 13 | 38.2 | 8.6 | 0.014* |
|  | IDK | 10 | 2 | 9 | 21 | 61.8 |  |  |

IDK: I do not know, PCC: Porcine cysticercosis, HCC: Human cysticercosis, * significant (p<0.05), %: percentage.
