# Supplementary material for: Lack of knowledge of stakeholders in the pork value chain: Considerations for transmission and control of Taenia solium and Toxoplasma gondii in Burundi
Source: PLoS One. 2025 Jul 2;20(7):e0326238. doi: 10.1371/journal.pone.0326238 (PMC12221015; doi:10.1371/journal.pone.0326238)
Supplement: S2 Table — (DOCX) [file pone.0326238.s005.docx]

**S2 Table. Knowledge of *T. solium* and *T. gondii* infections based on stakeholder groups**

| **Questions** | **Answers** | **Butchers** | **FSQCO** | **Pig farmers** | **Pig traders** | **Pork consumers** | **Slaughterhouse workers** | **Vets** | **Total** | **%** | χ^2^ | **P-value** |
| --- | --- | --- | --- | --- | --- | --- | --- | --- | --- | --- | --- | --- |
| Knowledge of *T. solium* cysts | Yes | 95 | 2 | 108 | 11 | 104 | 14 | 7 | 341 | 93.2 | 11.7 | 0.069 |
|  | No/IDK | 1 | 0 | 12 | 0 | 12 | 0 | 0 | 25 | 6.8 |  |  |
| Knowledge of the cause of PCC | Yes | 24 | 2 | 29 | 7 | 37 | 10 | 7 | 116 | 31.7 | 39.9 | <0.0001* |
|  | No/IDK | 72 | 0 | 91 | 4 | 79 | 4 | 0 | 250 | 68.3 |  |  |
| Knowledge of the cause of pork tapeworm | Yes | 23 | 2 | 52 | 4 | 42 | 3 | 7 | 133 | 37.9 | 24.1 | 0.0005* |
|  | No | 63 | 0 | 61 | 6 | 78 | 10 | 0 | 218 | 62.1 |  |  |
| Knowledge of the symptoms of pork tapeworm | Yes | 14 | 2 | 40 | 3 | 32 | 2 | 6 | 99 | 28.2 | 26.7 | 0.0001* |
|  | No | 72 | 0 | 73 | 7 | 88 | 11 | 1 | 252 | 71.8 |  |  |
| Knowledge of the cause of HCC | Yes | 0 | 1 | 3 | 0 | 2 | 0 | 5 | 11 | 78.6 | 6.9 | 0.076 |
|  | No | 0 | 0 | 0 | 0 | 3 | 0 | 0 | 3 | 21.4 |  |  |
| Knowledge of the symptoms of HCC | Yes | 0 | 1 | 3 | 0 | 4 | 0 | 5 | 13 | 92.9 | 1.9 | 0.58 |
|  | No | 0 | 0 | 0 | 0 | 1 | 0 | 0 | 1 | 7.1 |  |  |
| Knowledge of the cause of epilepsy | Yes | 22 | 2 | 10 | 5 | 30 | 10 | 7 | 86 | 22.3 | 68.3 | <0.0001* |
|  | No | 74 | 0 | 112 | 6 | 104 | 4 | 0 | 300 | 77.7 |  |  |
| Knowledge of the cause of toxoplasmosis | Yes | 0 | 1 | 8 | 0 | 0 | 0 | 4 | 13 | 38.2 | 11.4 | 0.043* |
|  | No | 2 | 0 | 8 | 0 | 8 | 1 | 2 | 21 | 61.8 |  |  |
| Knowledge of the symptoms of toxoplasmosis | Yes | 0 | 1 | 14 | 0 | 4 | 0 | 5 | 24 | 70.6 | 11.9 | 0.036* |
|  | No | 2 | 0 | 2 | 0 | 4 | 1 | 1 | 10 | 29.4 |  |  |

FSQCO: Food safety quality control officers, IDK: I do not know, PCC: Porcine cysticercosis, HCC: Human cysticercosis, * significant (p<0.05), χ^2^: Chi-square, %: percentage
