# Supplementary material for: Lack of knowledge of stakeholders in the pork value chain: Considerations for transmission and control of Taenia solium and Toxoplasma gondii in Burundi
Source: PLoS One. 2025 Jul 2;20(7):e0326238. doi: 10.1371/journal.pone.0326238 (PMC12221015; doi:10.1371/journal.pone.0326238)
Supplement: S3 Table — (DOCX) [file pone.0326238.s006.docx]

**S3 Table. Knowledge of *T. solium* and *T. gondii* infections based on the education level**

| **Questions** | **Answers** | **Elementary** | **Secondary** | **University** | **Total** | **%** | **Chi-square** | **P-value** |
| --- | --- | --- | --- | --- | --- | --- | --- | --- |
| Knowledge of *T. solium* cysts | Yes | 295 | 39 | 7 | 341 | 93.2 | 21.8 | <0.0001* |
|  | No/IDK | 13 | 11 | 1 | 25 | 6.8 |  |  |
| Knowledge of the cause of PCC | Yes | 92 | 19 | 5 | 116 | 31.7 | 4.9 | 0.086 |
|  | No | 216 | 31 | 3 | 250 | 68.3 |  |  |
| Knowledge of the cause of pork tapeworm | Yes | 97 | 28 | 8 | 133 | 31.7 | 16.6 | 0.0002* |
|  | No | 191 | 26 | 1 | 218 | 68.3 |  |  |
| Knowledge of the symptoms of pork tapeworm | Yes | 72 | 22 | 5 | 99 | 28.2 | 8.9 | 0.011* |
|  | No | 216 | 32 | 4 | 252 | 71.8 |  |  |
| Knowledge of the cause of HCC | Yes | 1 | 7 | 3 | 11 | 78.6 | 1.5 | 0.466 |
|  | No | 0 | 3 | 0 | 3 | 21.4 |  |  |
| Knowledge of the symptoms of HCC | Yes | 1 | 10 | 2 | 13 | 92.9 | 3.9 | 0.139 |
|  | No | 0 | 0 | 1 | 1 | 7.1 |  |  |
| Knowledge of the cause of epilepsy | Yes | 67 | 16 | 3 | 86 | 22.3 | 2.5 | 0.284 |
|  | No | 255 | 39 | 6 | 300 | 77.7 |  |  |
| Knowledge of the cause of toxoplasmosis | Yes | 3 | 8 | 2 | 13 | 38.2 | 4.6 | 0.099 |
|  | No | 10 | 11 | 0 | 21 | 61.8 |  |  |
| Knowledge of the symptoms of toxoplasmosis | Yes | 8 | 14 | 2 | 24 | 70.6 | 1.4 | 0.488 |
|  | No | 5 | 5 | 0 | 10 | 29.4 |  |  |

IDK: I do not know, PCC: Porcine cysticercosis, HCC: Human cysticercosis, * Significant (p<0.05), %: percentage.
