# Supplementary material for: Lack of knowledge of stakeholders in the pork value chain: Considerations for transmission and control of Taenia solium and Toxoplasma gondii in Burundi
Source: PLoS One. 2025 Jul 2;20(7):e0326238. doi: 10.1371/journal.pone.0326238 (PMC12221015; doi:10.1371/journal.pone.0326238)
Supplement: S4 Table — (DOCX) [file pone.0326238.s007.docx]

**S4 Table. Multivariate analysis using logistic regression model**

| **Variable** | **β-coefficients** | **P-value** |
| --- | --- | --- |
| **Knowledge about the cause of porcine cysticercosis** | | |
| Intercept | -1.75941 | <0.0001* |
| Location | 0.82089 | 0.002* |
| Age | 0.97856 | 0.0009* |
| Gender | -1.86843 | <0.0001* |
| Education | 0.53574 | 0.097 |
| Occupation | 0.00474 | 0.986 |
| Family size | 0.34845 | 0.191 |
| **Knowledge about the cause of pork tapeworm** | | |
| Intercept | -1.15562 | <0.0001* |
| Location | 1.03243 | <0.0001* |
| Age | -0.09973 | 0.705 |
| Gender | -0.70374 | 0.065 |
| Education | 1.05624 | 0.0003* |
| Occupation | -0.04195 | 0.871 |
| Family size | 0.39196 | 0.126 |
| **Knowledge about the cause of human cysticercosis** | | |
| Intercept | 77.121 | 1 |
| Location | -2.033 | 1 |
| Age | -48.523 | 1 |
| Gender | 45.859 | 1 |
| Education | -53.410 | 1 |
| Occupation | 98.992 | 1 |
| Family size | -47.615 | 1 |
| **Knowledge about the cause of epilepsy** | | |
| Intercept | -1.44897 | <0.0001* |
| Location | 0.38452 | 0.161 |
| Age | 0.37916 | 0.207 |
| Gender | -3.12738 | <0.0001* |
| Education | 0.54588 | 0.097 |
| Occupation | 0.11268 | 0.682 |
| Family size | -0.06398 | 0.827 |
| **Knowledge about the cause of toxoplasmosis** | | |
| Intercept | -17.1048 | 0.993 |
| Location | 1.2186 | 0.385 |
| Age | -0.7844 | 0.542 |
| Gender | -1.7222 | 0.081 |
| Education | 0.8515 | 0.462 |
| Occupation | 16.9485 | 0.994 |
| Family size | -0.4032 | 0.663 |
| **Practice for medical consultation for pork tapeworm infection** | | |
| Intercept | -1.62350 | <0.0001* |
| Location | 0.90207 | 0.0006* |
| Age | 0.54267 | 0.041* |
| Gender | 0.47850 | 0.072 |
| Education | -0.01019 | 0.974 |
| Occupation | -0.42040 | 0.110 |
| Family size | 0.17828 | 0.474 |
| **Practice for medical consultation for epilepsy** | | |
| Intercept | -1.56283 | <0.0001* |
| Location | 0.70793 | 0.004* |
| Age | 0.50686 | 0.041* |
| Gender | 0.10863 | 0.679 |
| Education | 0.30582 | 0.289 |
| Occupation | 0.29407 | 0.237 |
| Family size | 0.08739 | 0.716 |
| **Practice for medical consultation for toxoplasmosis** | | |
| Intercept | -5.4724 | <0.0001* |
| Location | 1.3844 | 0.036* |
| Age | 0.2024 | 0.695 |
| Gender | 0.9492 | 0.052 |
| Education | 2.8688 | <0.0001* |
| Occupation | 0.4632 | 0.510 |
| Family size | 0.1913 | 0.691 |
| **Practice for pork preparation** | | |
| Intercept | -1.8159 | <0.0001* |
| Location | -0.5372 | 0.052 |
| Age | 0.2621 | 0.328 |
| Gender | 1.2070 | <0.0001* |
| Education | 0.5033 | 0.117 |
| Occupation | 0.8714 | 0.060 |
| Family size | 0.3482 | 0.190 |
| **Knowledge about consequences of eating pork infected with cysts** | | |
| Intercept | -1.3851 | <0.0001* |
| Location | 1.3149 | <0.0001* |
| Age | 0.2654 | 0.316 |
| Gender | -0.9368 | 0.002* |
| Education | 0.8761 | 0.004* |
| Occupation | 0.1591 | 0.544 |
| Family size | 0.3639 | 0.156 |

*Significant (p<0.05)
