# Supplementary material for: Lack of knowledge of stakeholders in the pork value chain: Considerations for transmission and control of Taenia solium and Toxoplasma gondii in Burundi
Source: PLoS One. 2025 Jul 2;20(7):e0326238. doi: 10.1371/journal.pone.0326238 (PMC12221015; doi:10.1371/journal.pone.0326238)
Supplement: S5 Table — (DOCX) [file pone.0326238.s008.docx]

**S5 Table. Treatment-seeking practices and attitudes.**

| **Questions** | **Answers** | **Bujumbura** | **Kayanza** | **Ngozi** | **Total** | **%** | **Chi-square** | **p-value** |
| --- | --- | --- | --- | --- | --- | --- | --- | --- |
| Medical consultation for pork tapeworm | Yes | 39 | 51 | 26 | 116 | 30.1 | 51.1 | <0.0001* |
|  | No | 14 | 10 | 20 | 44 | 11.4 |  |  |
|  | IDK | 141 | 33 | 52 | 226 | 58.5 |  |  |
| If no, other routes for seeking treatment | Traditional medicine | 10 | 5 | 5 | 20 | 45.5 | 14.3 | 0.006* |
|  | Stay at home (poverty) | 2 | 5 | 15 | 22 | 50.0 |  |  |
|  | Pharmacy | 2 | 0 | 0 | 2 | 4.5 |  |  |
| Medical consultation for epilepsy | Yes | 51 | 52 | 38 | 141 | 36.5 | 25.9 | <0.0001* |
|  | No | 66 | 22 | 35 | 123 | 31.9 |  |  |
|  | IDK | 77 | 20 | 25 | 122 | 31.6 |  |  |
| Consultation for traditional healers | Yes | 14 | 1 | 3 | 18 | 7.3 | 65.6 | <0.0001* |
|  | No | 2 | 18 | 4 | 24 | 9.8 |  |  |
|  | IDK | 127 | 23 | 53 | 203 | 82.9 |  |  |
| Medical consultation for toxoplasmosis | Yes | 5 | 8 | 14 | 27 | 7.0 | 14.2 | 0.0008* |
|  | IDK | 189 | 86 | 84 | 359 | 93.0 |  |  |

IDK: I do not know, *significant (p<0.05), %: percentage.
