# Supplementary material for: Lack of knowledge of stakeholders in the pork value chain: Considerations for transmission and control of Taenia solium and Toxoplasma gondii in Burundi
Source: PLoS One. 2025 Jul 2;20(7):e0326238. doi: 10.1371/journal.pone.0326238 (PMC12221015; doi:10.1371/journal.pone.0326238)
Supplement: S6 Table — (DOCX) [file pone.0326238.s009.docx]

**S6 Table. Treatment-seeking routes based on stakeholder groups**

| **Questions** | **Answers** | **Butchers** | **FSQCO** | **Pig farmers** | **Pig traders** | **Pork consumers** | **Slaughterhouse workers** | **Vets** | **Total** | **%** | **χ^2^** | **P-value** |
| --- | --- | --- | --- | --- | --- | --- | --- | --- | --- | --- | --- | --- |
| Medical consultation for pork tapeworm | Yes | 17 | 0 | 51 | 2 | 40 | 3 | 3 | 116 | 30.1 | 27.5 | 0.006* |
|  | No | 7 | 0 | 13 | 2 | 21 | 0 | 1 | 44 | 11.4 |  |  |
|  | IDK | 72 | 2 | 58 | 7 | 73 | 11 | 3 | 226 | 58.5 |  |  |
| No medical consultation | Trad. medicine | 4 | 0 | 5 | 1 | 10 | 0 | 0 | 20 | 45.5 | 15.5 | 0.050 |
|  | Stay at home | 2 | 0 | 8 | 0 | 11 | 0 | 1 | 22 | 50.0 |  |  |
|  | Pharmacy | 1 | 0 | 0 | 1 | 0 | 0 | 0 | 2 | 4.5 |  |  |
| Medical consultation for epilepsy | Yes | 30 | 0 | 59 | 4 | 38 | 6 | 4 | 141 | 36.5 | 25.5 | 0.012* |
|  | No | 25 | 0 | 32 | 4 | 55 | 5 | 2 | 123 | 31.9 |  |  |
|  | IDK | 41 | 2 | 31 | 3 | 41 | 3 | 1 | 122 | 31.6 |  |  |
| Consultation for traditional healer | Yes | 1 | 0 | 2 | 2 | 13 | 0 | 0 | 18 | 7.3 | 27.9 | 0.005* |
|  | No | 2 | 0 | 12 | 0 | 10 | 0 | 0 | 24 | 9.8 |  |  |
|  | IDK | 63 | 2 | 49 | 5 | 73 | 8 | 3 | 203 | 82.9 |  |  |
| Medical consultation for toxoplasmosis | Yes | 1 | 1 | 16 | 0 | 4 | 0 | 5 | 27 | 7.0 | 67.8 | <0.0001* |
|  | No/IDK | 95 | 1 | 106 | 11 | 130 | 14 | 2 | 359 | 93.0 |  |  |

FSQCO: Food safety quality control officers, IDK: I do not know, χ^2^: Chi-square, *Significant (p<0.05), %: percentage.
