# Supplementary material for: Lack of knowledge of stakeholders in the pork value chain: Considerations for transmission and control of Taenia solium and Toxoplasma gondii in Burundi
Source: PLoS One. 2025 Jul 2;20(7):e0326238. doi: 10.1371/journal.pone.0326238 (PMC12221015; doi:10.1371/journal.pone.0326238)
Supplement: S7 Table — (DOCX) [file pone.0326238.s010.docx]

**S7 Table. Treatment-seeking routes based on the education level**

| **Questions** | **Answers** | **Elementary** | **Secondary** | **University** | **Total** | **%** | **Chi-square** | **P-value** |
| --- | --- | --- | --- | --- | --- | --- | --- | --- |
| Medical consultation for pork tapeworm | Yes | 97 | 16 | 3 | 116 | 30.1 | 1.7 | 0.79 |
|  | No | 39 | 5 | 0 | 44 | 11.4 |  |  |
|  | IDK | 186 | 34 | 6 | 226 | 58.5 |  |  |
| No medical consultation | Trad. medicine | 18 | 2 | 0 | 20 | 45.5 | 0.4 | 0.82 |
|  | Stay at home | 19 | 3 | 0 | 22 | 50.0 |  |  |
|  | Pharmacy | 2 | 0 | 0 | 2 | 4.5 |  |  |
| Medical consultation for epilepsy | Yes | 114 | 23 | 4 | 141 | 36.5 | 9.6 | 0.048* |
|  | No | 112 | 11 | 0 | 123 | 31.9 |  |  |
|  | IDK | 96 | 21 | 5 | 122 | 31.6 |  |  |
| Consultation for traditional healer | Yes | 16 | 2 | 0 | 18 | 7.3 | 1.8 | 0.78 |
|  | No | 22 | 2 | 0 | 24 | 9.8 |  |  |
|  | IDK | 160 | 28 | 5 | 203 | 82.9 |  |  |
| Medical consultation for toxoplasmosis | Yes | 9 | 16 | 2 | 27 | 7.0 | 53.2 | <0.0001* |
|  | No/IDK | 313 | 39 | 7 | 359 | 93.0 |  |  |

IDK: I do not know, %: Percentage, * Significant (p<0.05)
