# Supplementary material for: Lack of knowledge of stakeholders in the pork value chain: Considerations for transmission and control of Taenia solium and Toxoplasma gondii in Burundi
Source: PLoS One. 2025 Jul 2;20(7):e0326238. doi: 10.1371/journal.pone.0326238 (PMC12221015; doi:10.1371/journal.pone.0326238)
Supplement: S8 Table — (DOCX) [file pone.0326238.s011.docx]

**S8 Table. Practices for pork consumption.**

| **Questions** | **Answers** | **Bujumbura** | **Kayanza** | **Ngozi** | **Total** | **%** | **χ^2^** | **p-value** |
| --- | --- | --- | --- | --- | --- | --- | --- | --- |
| Eating pork | Yes | 192 | 86 | 83 | 361 | 93.5 | 22.7 | <0.0001* |
|  | No | 2 | 8 | 15 | 25 | 6.5 |  |  |
| Frequency of eating pork | Daily | 93 | 14 | 10 | 117 | 32.4 | 132.9 | <0.0001* |
|  | During a week | 88 | 15 | 32 | 135 | 37.4 |  |  |
|  | During a month | 7 | 18 | 15 | 40 | 11.1 |  |  |
|  | Occasionally a year | 4 | 39 | 26 | 69 | 19.1 |  |  |
| Preference for pork to be prepared | Roasting | 154 | 59 | 56 | 269 | 74.5 | 34.3 | <0.0001* |
|  | Boiling | 12 | 9 | 23 | 44 | 12.2 |  |  |
|  | Boiling and frying | 15 | 13 | 4 | 32 | 8.9 |  |  |
|  | Frying | 11 | 5 | 0 | 16 | 4.4 |  |  |
| Quantity of pork consumed | 100-200g | 107 | 79 | 69 | 255 | 70.6 | 45.5 | <0.0001* |
|  | >200-500g | 85 | 7 | 14 | 106 | 29.4 |  |  |
| Beliefs of eating pigs infected with cysts | Yes | 172 | 85 | 93 | 350 | 90.7 | 3.0 | 0.223 |
|  | No | 22 | 9 | 5 | 36 | 9.3 |  |  |
| Consequences of eating pork infected with cysts | Taeniosis | 28 | 46 | 37 | 111 | 31.7 | 43.4 | <0.0001* |
|  | Epilepsy | 20 | 8 | 7 | 35 | 10.0 |  |  |
|  | Illnesses | 120 | 29 | 47 | 196 | 56.0 |  |  |
|  | IDK | 4 | 2 | 2 | 8 | 2.3 |  |  |

IDK: I do not know, * significant (<0.05), %: percentage, χ^2^: Chi-square
