# Supplementary material for: Lack of knowledge of stakeholders in the pork value chain: Considerations for transmission and control of Taenia solium and Toxoplasma gondii in Burundi
Source: PLoS One. 2025 Jul 2;20(7):e0326238. doi: 10.1371/journal.pone.0326238 (PMC12221015; doi:10.1371/journal.pone.0326238)
Supplement: S9 Table — (DOCX) [file pone.0326238.s012.docx]

**S9 Table. Pork consumption and preparation based on stakeholder groups**

| **Questions** | **Answers** | **Butchers** | **FSQCO** | **Pig farmers** | **Pig traders** | **Pork consumers** | **Slaughterhouse workers** | **Vets** | **Total** | **%** | **Chi-square** | **p-value** |
| --- | --- | --- | --- | --- | --- | --- | --- | --- | --- | --- | --- | --- |
| Eating pork | Yes | 96 | 0 | 100 | 11 | 134 | 14 | 6 | 361 | 93.5 | 74.1 | <0.0001* |
|  | No | 0 | 2 | 22 | 0 | 0 | 0 | 1 | 25 | 6.5 |  |  |
| Pork preparation ways | Roasting | 86 | 0 | 49 | 5 | 115 | 10 | 4 | 269 | 74.5 | 31.7 | <0.0001* |
|  | Cooking/frying | 10 | 0 | 51 | 6 | 19 | 4 | 2 | 92 | 25.5 |  |  |
| Bad behaviour of eating infected pork | Yes | 87 | 2 | 110 | 12 | 120 | 14 | 7 | 350 | 90.7 | 2.6 | 0.856 |
|  | No | 9 | 0 | 12 | 1 | 14 | 0 | 0 | 36 | 9.3 |  |  |
| Consequences of eating infected pork | Taeniosis | 18 | 2 | 51 | 1 | 32 | 0 | 7 | 111 | 31.7 | 93.6 | <0.0001* |
|  | Epilepsy | 9 | 0 | 4 | 3 | 10 | 9 | 0 | 35 | 10.0 |  |  |
|  | Illnesses | 58 | 0 | 51 | 6 | 76 | 5 | 0 | 196 | 56.0 |  |  |
|  | IDK | 2 | 0 | 4 | 0 | 2 | 0 | 0 | 8 | 2.3 |  |  |

FSQCO: Food safety quality control officers, IDK: I do not know, *Significant (p<0.05), %: percentage.
