# Supplementary material for: Lack of knowledge of stakeholders in the pork value chain: Considerations for transmission and control of Taenia solium and Toxoplasma gondii in Burundi
Source: PLoS One. 2025 Jul 2;20(7):e0326238. doi: 10.1371/journal.pone.0326238 (PMC12221015; doi:10.1371/journal.pone.0326238)
Supplement: S10 Table — (DOCX) [file pone.0326238.s013.docx]

**S10 Table. Pork consumption and preparation based on the education level**

| **Questions** | **Answers** | **Elementary** | **Secondary** | **University** | **Total** | **%** | **Chi-square** | **P-value** |
| --- | --- | --- | --- | --- | --- | --- | --- | --- |
| Eating pork | Yes | 306 | 50 | 5 | 361 | 93.5 | 23.2 | <0.0001* |
|  | No | 16 | 5 | 4 | 25 | 6.5 |  |  |
| Pork preparation ways | Roasting | 232 | 35 | 2 | 269 | 74.5 | 3.9 | 0.139 |
|  | Cooking/frying | 74 | 15 | 3 | 92 | 25.5 |  |  |
| Bad behaviour of eating infected pork | Yes | 287 | 54 | 9 | 350 | 90.7 | 5.5 | 0.064 |
|  | No | 35 | 1 | 0 | 36 | 9.3 |  |  |
| Consequences of eating infected pork | Taeniosis | 76 | 28 | 7 | 111 | 31.7 | 25.5 | 0.0003* |
|  | Epilepsy | 34 | 1 | 0 | 35 | 10.0 |  |  |
|  | Illnesses | 169 | 25 | 2 | 196 | 56.0 |  |  |
|  | IDK | 8 | 0 | 0 | 8 | 2.3 |  |  |

IDK: I do not know, %: percentage, *Significant (p<0.05).
