# Supplementary material for: Lack of knowledge of stakeholders in the pork value chain: Considerations for transmission and control of Taenia solium and Toxoplasma gondii in Burundi
Source: PLoS One. 2025 Jul 2;20(7):e0326238. doi: 10.1371/journal.pone.0326238 (PMC12221015; doi:10.1371/journal.pone.0326238)
Supplement: Attachment — Submitted filename: PONE-D-24-40420_ReviewReport.docx [file pone.0326238.s014.docx]

PONE-D-24-40420

TITLE

Lack of knowledge of stakeholders in the pork value chain: considerations for transmission and control of *Taenia solium* and *Toxoplasma gondii* in Burundi

**Review Report**

This paper reports the level of knowledge, health-seeking behaviour and risk factors for prevalence of infections with *Taenia solium* and *Toxoplasma gondii* in Burundi. The researchers used a mixed-method approach to capture as much information as possible and validate it through triangulation of the information. This article makes significant contribution to the existing body of knowledge in these neglected parasitic zoonoses in Burundi and Sub-Saharan Africa at large.

To improve the reporting of this piece of research, the authors should consider the following comments based on my review of the manuscript:

ABSTRACT

- Despite the use of a combination of quantitative, qualitative, and observational methods of data collection, the abstract reports only qualitative information. I advise the authors to report some key quantitative results in the abstract as well.
- The sample size information for the qualitative study should also be provided in the abstract.
- Results provided in the abstract should indicate their respective source/basis (quantitative versus qualitative study).

INTRODUCTION

The authors should provide information on the prevalence of *Taenia solium* and *Toxoplasma gondii* in Burundi. Currently, no mention at all. If they are not prevalent, the current study lacks a foundation.

MATERIALS AND METHODS

**Quantitative strand - sampling and data collection**

- The sample size formula for the quantitative study provided is not elaborated. Authors should (i) define the letters included in the formula and (ii) the values they have used to arrive at the 386 people.
- The authors said “All interview data were validated before analysis”. They should explain how the validation was done.
- Could running a multivariate analysis add value to the research findings?

**Qualitative strand**

- Determination of sample sizes for the various components (FGDs, informal conversation, and observations) is not described.
- Qualitative data analysis is not clearly described.

RESULTS

- Authors should double-check the conformance of the manuscript with the PLOSONE format requirement. I find specifically that the vertical and several lines in the tables might not conform to the journal’s format. Similarly, some tables are too long.
- As for porcine cysticercosis, please mention the local name for toxoplasmosis.
