# Supplementary material for: Lack of knowledge of stakeholders in the pork value chain: Considerations for transmission and control of Taenia solium and Toxoplasma gondii in Burundi
Source: PLoS One. 2025 Jul 2;20(7):e0326238. doi: 10.1371/journal.pone.0326238 (PMC12221015; doi:10.1371/journal.pone.0326238)
Supplement: Attachment — Submitted filename: Response to Reviewers.docx [file pone.0326238.s015.docx]

**Responses to Reviewer comments**

PONE-D-24-40420

**TITLE**

Lack of knowledge of stakeholders in the pork value chain: considerations for transmission and control of *Taenia solium* and *Toxoplasma gondii* in Burundi

**Review Report**

This paper reports the level of knowledge, health-seeking behaviour and risk factors for prevalence of infections with *Taenia solium* and *Toxoplasma gondii* in Burundi. The researchers used a mixed-method approach to capture as much information as possible and validate it through triangulation of the information. This article makes significant contribution to the existing body of knowledge in these neglected parasitic zoonoses in Burundi and Sub-Saharan Africa at large.

Thank you very much for the kind appreciation

To improve the reporting of this piece of research, the authors should consider the following comments based on my review of the manuscript:

**ABSTRACT**

Despite the use of a combination of quantitative, qualitative, and observational methods of data collection, the abstract reports only qualitative information. I advise the authors to report some key quantitative results in the abstract as well.

Thank you for your comments. This was corrected as suggested.

- The sample size information for the qualitative study should also be provided in the abstract. This was added as suggested (Line 25).
- Results provided in the abstract should indicate their respective source/basis (quantitative versus qualitative study). This was added as suggested (Lines 26-36).

**INTRODUCTION**

The authors should provide information on the prevalence of *Taenia solium* and *Toxoplasma gondii* in Burundi. Currently, no mention at all. If they are not prevalent, the current study lacks a foundation. This was added (Lines 57-59, Lines 74-75).

**MATERIALS AND METHODS**

**Quantitative strand - sampling and data collection**

- The sample size formula for the quantitative study provided is not elaborated. Authors should (i) define the letters included in the formula and (ii) the values they have used to arrive at the 386 people. This was corrected as suggested (Lines 128-133).

The authors said “All interview data were validated before analysis”. They should explain how the validation was done. This was explained as suggested (Lines 159-161).

- Could running a multivariate analysis add value to the research findings? This was done as suggested (Lines 164-170, 267-269, 312-315, 340-341, 351-353, 383-384, 407-409, 425-427, 440-443, 496-497, 525-528).

**Qualitative strand**

- Determination of sample sizes for the various components (FGDs, informal conversation, and observations) is not described.

We do think that the description was sufficiently done because qualitative research focuses on the quality of data obtained rather than determining a fixed sample size for participants. Data saturation is a key indicator for stopping participant recruitment. We ensured that all dimensions of the research were covered and that data saturation was reached (Lines 176-178). In this study, we conducted a qualitative study to triangulate and compare data from the quantitative study. Thus, we designed FGDs in three study areas (Bujumbura city, Kayanza, and Ngozi), with each FGD including 8 or 9 participants. Since data saturation is a key indicator in qualitative research, two FGDs per study area were sufficient, resulting in a total of six FGDs (Lines 183-185, Lines 190-191). To complement the data from FGDs and interviews (quantitative study), we conducted informal conversations and observations to better understand the local context and practices in pig farms, slaughterhouses, pork outlets, and pork consumption, which could be potential risk factors for T. solium and T. gondii transmission in humans and pigs (Lines 193-197). This is why we did not specify a sample size for FGD, informal conversations, and observations.

- Qualitative data analysis is not clearly described. This has been added and is now clearly described as suggested (Lines 199-206).

**RESULTS**

- Authors should double-check the conformance of the manuscript with the PLOSONE format requirement. I find specifically that the vertical and several lines in the tables might not conform to the journal’s format. Similarly, some tables are too long. This was checked and corrected. All long tables were moved to the supporting information.
- As for porcine cysticercosis, please mention the local name for toxoplasmosis. The local name for toxoplasmosis was missing, as the disease is largely unknown within the community. Those who were aware of it had heard about it during visits to health facilities or prenatal consultations at hospitals. At the hospital, all medical examinations are written in French, where toxoplasmosis is referred to as 'toxoplasmose' and abbreviated as 'toxo,' which is not easily understood by everyone.
